# Analysis of Transcriptional Regulatory Pathways of Photoreceptor Genes by Expression Profiling of the *Otx2-*Deficient Retina

**DOI:** 10.1371/journal.pone.0019685

**Published:** 2011-05-13

**Authors:** Yoshihiro Omori, Kimiko Katoh, Shigeru Sato, Yuki Muranishi, Taro Chaya, Akishi Onishi, Takashi Minami, Takashi Fujikado, Takahisa Furukawa

**Affiliations:** 1 Department of Developmental Biology, Osaka Bioscience Institute, Osaka, Japan; 2 JST, CREST, Osaka, Japan; 3 PRESTO, Suita, Osaka, Japan; 4 Department of Ophthalmology, Osaka University Graduate School of Medicine, Suita, Osaka, Japan; 5 Department of Visual Science, Osaka University Graduate School of Medicine, Suita, Osaka, Japan; 6 The Research Center for Advanced Science and Technology, The University of Tokyo, Tokyo, Japan; University of Oldenburg, Germany

## Abstract

In the vertebrate retina, the Otx2 transcription factor plays a crucial role in the cell fate determination of both rod and cone photoreceptors. We previously reported that *Otx2* conditional knockout (CKO) mice exhibited a total absence of rods and cones in the retina due to their cell fate conversion to amacrine-like cells. In order to investigate the entire transcriptome of the *Otx2* CKO retina, we compared expression profile of *Otx2* CKO and wild-type retinas at P1 and P12 using microarray. We observed that expression of 101- and 1049-probe sets significantly decreased in the *Otx2* CKO retina at P1 and P12, respectively, whereas, expression of 3- and 4149-probe sets increased at P1 and P12, respectively. We found that expression of genes encoding transcription factors involved in photoreceptor development, including *Crx, Nrl, Nr2e3, Esrrb,* and *NeuroD*, was markedly down-regulated in the *Otx2* CKO at both P1 and P12. Furthermore, we identified three human retinal disease loci mapped in close proximity to certain down-regulated genes in the *Otx2* CKO retina including *Ccdc126, Tnfsf13* and *Pitpnm1*, suggesting that these genes are possibly responsible for these diseases. These transcriptome data sets of the *Otx2* CKO retina provide a resource on developing rods and cones to further understand the molecular mechanisms underlying photoreceptor development, function and disease.

## Introduction

During mammalian retinogenesis, five major types of neurons arise from multipotent progenitor cells, which are common precursors for all retinal neurons and glia [1], [2], [3]. We previously demonstrated that *Otx2*, an *Otx-*like homeobox gene, is essential for the cell fate determination of retinal photoreceptor cells [4]. *Otx2* conditional knockout (CKO) mice showed a cell fate switch from retinal photoreceptor precursor cells to amacrine-like cells. On the other hand, several transcription factors including *Crx*, *Nrl* and *Nr2e3* are essential for terminal differentiation of photoreceptors. *Crx* encodes an *Otx-*like homeodomain transcription factor essential for terminal differentiation of both rods and cones by regulating genes encoding phototransduction, photoreceptor metabolism and outer segment formation [5], [6]. *Crx* knockout (KO) mice develop aberrant photoreceptors that lack both rod and cone photoresponses [7]. In humans, mutations in *CRX* are associated with retinal degeneration diseases such as cone-rod dystrophy-2, retinitis pigmentosa (RP), and Leber congenital amaurosis (LCA) [6], [8], [9], [10]. Nrl (neural retina leucine zipper gene) is a transcription factor of the leucine zipper family expressed predominantly in rods but not in cones [11]. Mice lacking the *Nrl* gene do not develop rods but produce an increased number of short wavelength-sensitive cones (S-cones) [12]. Nrl promotes rod development by directly activating rod-specific genes while simultaneously suppressing the S-cone related genes through the activation of transcriptional repressor Nuclear receptor subfamily 2 group E member 3 (Nr2e3) [12]. Mutations in human *NR2E3* cause enhanced S-cone syndrome [13]. The *rd7* mouse has a genetic defect in the *Nr2e3* gene and exhibits an increased number of cones [14]. Mice lacking retinoid-related orphan nuclear receptor β (*Rorb*) were shown to lose rods but overproduce primitive S-cones, similar to *Nrl* KO mice [15]. In addition, several other nuclear receptors are involved in both photoreceptor development and transcriptional regulation of photoreceptor-specific genes. During the terminal differentiation of cone photoreceptors, thyroid hormone receptor β2 (Trb2) is critical for M-opsin induction [16], whereas retinoid X receptor γ (Rxrg) is essential for suppressing S-opsin in cone photoreceptors [17]. Retinoic acid receptor-related orphan receptor α (Rora) directly controls expression of cone opsins and arrestin3 [18]. In rod photoreceptor cells, estrogen-related receptor β (Esrrb) regulates expression of rod-specific genes and controls rod photoreceptor survival [19]. Recently, Pias3, an E3 SUMO ligase which is selectively expressed in developing photoreceptors, was shown to SUMOylate Nr2e3 and promote the differentiation of rod-photoreceptors [20]. In addition, Pias3 regulates expression of cone opsins by modulating Rxrg, Rora, and Trb1 [21].

During the terminal differentiation of photoreceptors, the photoreceptor axon terminal develops a highly specialized synapse, the ribbon synapse, which connects photoreceptor axonal terminals with bipolar and horizontal dendritic terminals in the outer plexiform layer (OPL) of the retina [22]. The functional ribbon synapse structure is organized by the precise assembly of presynaptic components including CtBP2, bassoon, pikachurin, CaBP4 and Cacna1f [23], [24], [25], [26]. Photoreceptor cells develop the photosensitive outer segments which contain molecules involved in phototransduction, such as opsins and transducins, and outer segment morphogenesis factors, such as Rom1 and Peripherin2 [27]. Outer segments are formed from the primary cilia [28]. In humans disruption of photoreceptor ciliary function causes retinal diseases including retinitis pigmentosa, Bardet-Biedl syndrome (BBS) and Nephronophthisis (NPHP) [27], [29], [30]. Mutations in genes encoding ciliary components including Rpgrip1, Rp1, and Mak cause photoreceptor degeneration and retinal dysfunction in mice [31], [32], [33].

To our knowledge, the *Otx2* CKO mouse is the only mutant which shows defects of both rods and cones in the retina from early developmental stages. In this study, we investigated the transcriptional profile of both developing rods and cones by taking advantage of the *Otx2* CKO retina.

## Results

### Identification of differentially expressed genes in the *Otx2* CKO retina

In order to clarify the molecular role of *Otx2* in transcriptional regulation during development, we investigated the expression profile of the *Otx2* CKO retina compared with that of the control retina with the genotype *Otx2^flox/flox^/Crx-cre^-^* using microarrays at two time points, postnatal day 1 (P1) and P12. From middle late embryonic stages cones are generated with almost all of the cones formed by P0 [34]. Rods begin to form in embryonic stages whereas, photoreceptor maturation, including ribbon synapse and outer segment formation, occurs in P0 to P14 [35], [36]. By P12, all photoreceptor cells are born, however, photoreceptor maturation, such as outer segment formation, is ongoing. Thus, we expect that the expression profile at P1 will mainly reflect cone development and early rod development, and the expression profile at P12 will mainly reflect rod development.

We performed genome-wide expression profiling using a microarray containing 45,101 probe sets covering more than 34,000 genes (Mouse Genome 430 2.0; Affymetrix). In the *Otx2* CKO retina at P1, we identified 101 down-regulated probes (signal log ratio ≤−1.0, signal intensity ≥50) and 3 up-regulated probes (signal log ratio ≥+1.0, signal intensity ≥50) compared to the control retina at P1. In the *Otx2* CKO retina at P12, we identified 1049 down-regulated probes (signal log ratio ≤−1.0, signal intensity ≥50) and 4149 up-regulated probes (signal log ratio ≥+1.0, signal intensity ≥50) compared to the control retina at P12.

**Figure 1:**
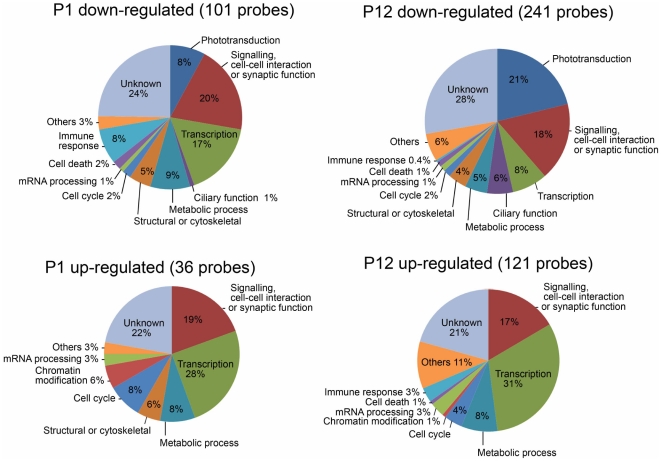
Categorization of the genes differentially expressed in the *Otx2* CKO retina. According to gene ontology (GO) term annotation, the genes differentially expressed in the *Otx2* CKO retinas at P1 and P12 were categorized into functional groups. We used GO term data on 101 probes with decreased expression in the *Otx2* CKO at P1 (signal log ratio ≤−1.0, signal intensity ≥50), 36 probes with increased expression in the *Otx2* CKO at P1 (signal log ratio ≥+0.5, signal intensity ≥50), 241 probes with decreased expression in the *Otx2* CKO at P12 (signal log ratio ≤−2.5, signal intensity ≥100), and 121 probes with increased expression in the *Otx2* CKO at P12 (signal log ratio ≥+2.5, signal intensity ≥100).

To compare the features of these groups of probe sets, we categorized functions of these genes based on their gene ontology (GO) term annotations (Fig. 1). Genes involved in “phototransduction” and “ciliary function” in the photoreceptor cells were observed in the groups with down-regulated expression in the *Otx2* CKO retina at both P1 and P12. The proportion of probe sets involved in these categories increased in the P12 down-regulated group (21% for phototransduction, 6% for ciliary function) compared to P1 down-regulated group (8% for phototransduction, 1% for ciliary function). We found that the proportion of genes categorized in “cell cycle” and “transcription” in the up-regulated probes in the *Otx2* CKO retina was higher than that in down-regulated probes both at P1 and P12.

**Figure 2:**
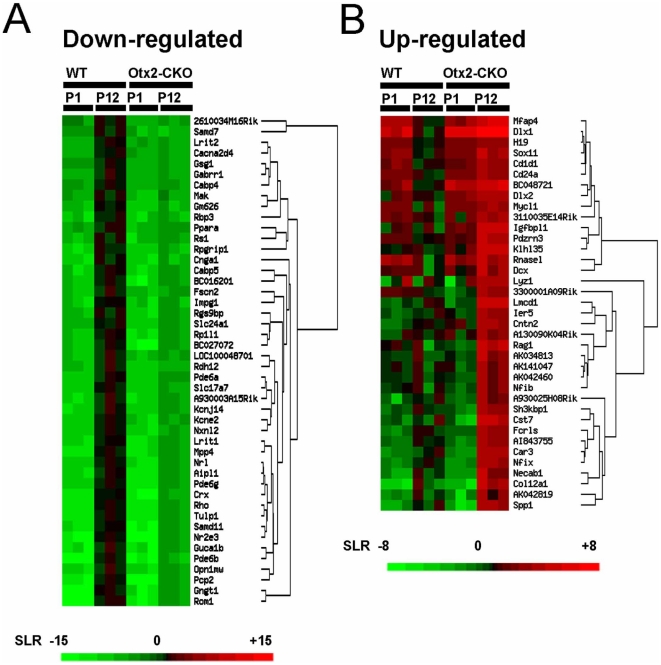
Clustering analysis of retinal gene expression profiles in the *Otx2* CKO and control retinas. (A, B) Matrices were visualized as hierarchical clustering of down-regulated (A) and up-regulated (B) genes in the *Otx2* CKO retina at P1 and P12. Each gene was visualized as a single row of colored squares. The color indicates the relative expression level of a gene. Signal log ratio (SLR) was calculated based on the average of signal intensity of the control retina at P12. The color scale ranges are from signal log ratios of −15 to +15 (A) and −8 to +8 (B) as shown in the scale bar on the bottom of the figure. Gene symbols are indicated in the lists on the right of the matrixes. Hierarchical clustering was performed by the EPCLUST program (http://www.bioinf.ebc.ee/EP/EP/EPCLUST/).

**Figure 3:**
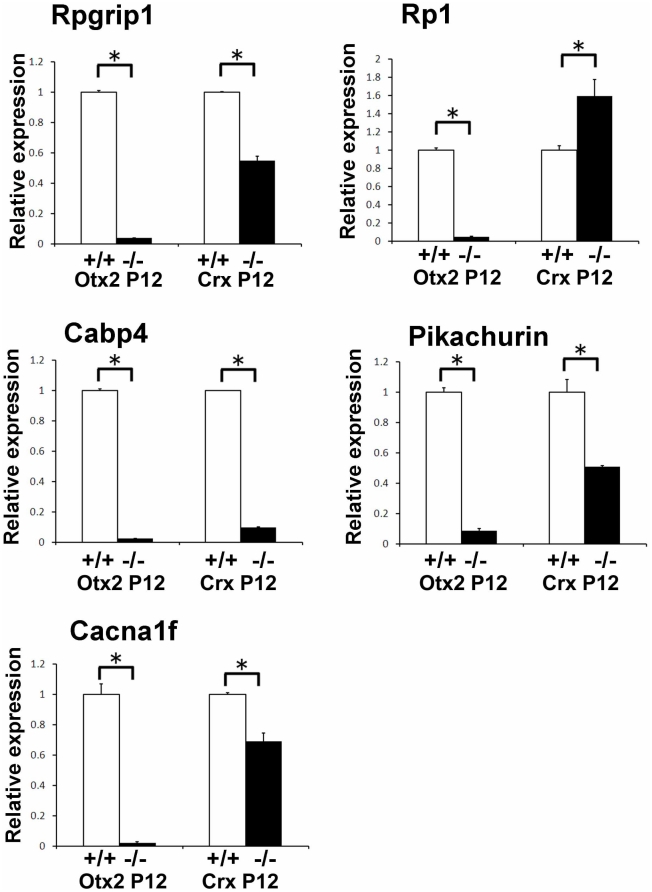
Expression of genes encoding photoreceptor ciliary and ribbon synaptic components in the *Otx2* CKO and *Crx* KO retina. Expression levels of genes encoding photoreceptor ciliary and ribbon synaptic components in the control, *Otx2* CKO and *Crx* KO retinas at P12 were analyzed by Q-PCR. Expression levels of selected genes were normalized to the expression levels of a housekeeping gene, *Gapdh*. Primer sequences for PCR were shown in Table S1. The mean of the value of control at P12 was set as 1.0. Error bars show the SD (n = 3). *, P<0.03.

Using the data from three independent microarray experiments at P1 and P12, we performed hierarchical clustering analysis on 46 strongly down-regulated genes (signal log ratio ≤−5.0, signal intensity ≥100, Table 1) and 37 up-regulated genes (signal log ratio ≥+3.0, signal intensity ≥100, Table 2) in the *Otx2* CKO retina at P12 (Fig. 2). Expression levels of all of strongly down-regulated genes were more than 10 times higher in the P12 control retina compared to those of P1. Most of these down-regulated genes encode known photoreceptor-associated genes including transcription factors (*Crx, Nrl, Nr2e3*), and phototransduction molecules (*Rho, Opn1mw, Pde6a, Pde6b, Cnga1*) (Fig. 2, Table 1). We also confirmed that the genes encoding photoreceptor ciliary and ribbon synaptic components including Rpgrip1, Rp1, Cabp4, Pikachurin, and Cacna1f showed drastically decreased expression both in microarray and Q-PCR analysis (Fig. 3).

**Table 1:** Genes down-regulated in the *Otx2* CKO retina (signal log ratio ≤−5.0).

Probe ID	Gene symbol	P1 WT	P12 WT	P1 CKO	P12 CKO	SLR	Cell Type	*Nrl* KO
1430817_at	Samd7	12.4	221.6	1.9	2.0	−6.8	Photoreceptor	
1431010_a_at	Rdh12	2.2	1411.2	0.4	19.9	−6.2	Photoreceptor	
1457855_at	Rbp3	8.4	190.0	1.2	2.7	−6.1	Photoreceptor	
1429133_at	Nxnl2	48.6	1742.8	2.4	31.3	−5.8	Photoreceptor	
1458506_at	Gm626/Frmpd2	10.8	962.8	1.7	19.1	−5.7		
1451785_at	Rpgrip1	27.6	653.9	3.8	13.0	−5.6	Photoreceptor	
1440605_at	Fscn2	0.3	128.2	1.0	2.6	−5.6	Rod	down
1425757_a_at	Impg1	1.6	266.2	1.5	5.4	−5.6	Photoreceptor	
1419740_at	Pde6b	16.4	4001.8	1.2	83.8	−5.6	Rod	down
1425306_at	BC027072/C2orf71	26.6	1241.9	2.2	26.1	−5.6		
1423631_at	Nr2e3	95.0	2540.0	7.2	57.4	−5.5	Rod	down
1428986_at	Slc17a7	3.0	1384.0	2.6	32.0	−5.4	Photoreceptor	
1444095_a_at	LOC100048701	1.2	469.3	0.5	11.4	−5.4		
1443940_at	Lrit2	9.9	363.7	1.7	8.9	−5.4	Photoreceptor	
1456936_at	Cabp4	2.8	569.7	0.8	14.1	−5.3	Photoreceptor	
1451582_at	Tulp1	98.3	3258.0	10.6	80.6	−5.3	Photoreceptor	
1452243_at	Kcnj14	1.5	708.9	0.3	17.7	−5.3	Rod	down
1425288_at	Samd11	67.5	1913.3	11.4	48.2	−5.3	Rod	down
1451826_at	Cabp5	6.4	774.2	1.0	19.8	−5.3	Bipolar	up
1419723_at	Opn1mw	0.9	847.4	2.0	22.1	−5.3	Cone	
1419085_at	Pcp2	13.4	2792.9	1.5	73.6	−5.2	Bipolar	
1441110_at	Lrit1	14.5	1111.4	1.4	30.3	−5.2	Photoreceptor	
1421084_at	Rs1	1.8	755.8	0.4	20.9	−5.2	Photoreceptor	
1425138_at	Guca1b	4.0	1787.6	1.0	49.8	−5.2	Rod	down
1451549_at	Cngb1	7.8	1634.8	2.6	45.6	−5.2	Rod	down
1450300_at	Gabrr1	2.2	547.3	2.2	15.3	−5.2	Photoreceptor	
1449051_at	Ppara	1.0	109.9	1.3	3.1	−5.2		
1427952_at	Aipl1	22.8	2769.5	4.0	78.0	−5.1	Cone	
1451763_at	Cnga1	1.7	687.8	0.6	19.4	−5.1	Rod	down
1451617_at	Rho	6.8	5886.7	2.9	167.5	−5.1	Rod	down
1455203_at	A930003A15Rik	11.3	1176.5	4.3	33.9	−5.1		
1425408_a_at	2610034M16Rik	0.9	117.8	0.7	3.4	−5.1		down
1450453_a_at	Pde6g	26.3	4870.2	5.6	140.8	−5.1	Rod	
1457622_at	Rp1l1	15.5	1055.1	2.4	30.7	−5.1	Rod	
1442863_at	Cacna2d4	11.5	306.6	1.5	9.0	−5.1	Photoreceptor	
1460368_at	Mpp4	18.3	2727.2	3.6	80.9	−5.1	Photoreceptor	
1425219_x_at	Gngt1	6.2	2929.2	1.6	87.2	−5.1	Photoreceptor	
1451647_at	Slc24a1	1.7	1526.9	1.5	45.5	−5.1	Rod	down
1448996_at	Rom1	76.8	3650.3	6.4	112.0	−5.0	Rod	
1449421_a_at	Kcne2	3.0	452.8	0.6	14.0	−5.0	Cone	up
1417550_a_at	Gsg1	2.1	338.6	1.0	10.5	−5.0		
1449185_at	Crx	159.1	2167.9	12.3	68.2	−5.0	Photoreceptor	
1440256_at	Rgs9bp	6.9	691.1	1.2	21.8	−5.0	Photoreceptor	
1450946_at	Nrl	293.9	6073.3	18.6	191.9	−5.0	Rod	down
1450415_at	Pde6a	2.0	728.5	0.7	23.2	−5.0	Rod	down
1422724_at	Mak	18.5	429.6	1.5	13.8	-5.0	Photoreceptor	

**Table 2:** Genes up-regulated in the *Otx2* CKO retina (signal log ratio ≥+3.0).

Probe Set ID	Gene Symbol	P1 WT	P12 WT	P1 CKO	P12 CKO	P12/P1	P12WT/CKO	Cell type
1449470_at	Dlx1	26.4	0.7	66.0	273.4	−5.2	8.6	GCL(d)
1424010_at	Mfap4	23.7	1.5	10.5	102.5	−4.0	6.1	
1450960_at	Igfbpl1	13.6	10.9	26.3	445.7	−0.3	5.3	
1431225_at	BC048721	339.0	42.9	826.0	1691.1	−3.0	5.3	
1424596_s_at	Lmcd1	1.5	3.0	1.4	108.6	1.0	5.2	
1450680_at	Rag1	6.3	6.0	3.0	180.6	−0.1	4.9	
1429945_at	Klhl35	17.4	16.8	33.4	390.2	0.0	4.5	
1419202_at	Cst7	2.0	4.5	1.3	101.0	1.2	4.5	
1434411_at	Col12a1	0.2	10.3	0.8	220.8	5.5	4.4	
1426604_at	Rnasel	103.1	11.9	90.2	234.4	−3.1	4.3	PR+INL(B)
1416846_a_at	Pdzrn3	106.1	55.7	103.4	878.1	−0.9	4.0	
1439426_x_at	Lyz1	50.6	11.9	18.5	182.9	−2.1	3.9	
1453125_at	Sox11	344.6	60.7	255.6	845.5	−2.5	3.8	PR+INL(B)
1438245_at	AK034813	77.5	96.7	68.2	1295.1	0.3	3.7	
1418139_at	Dcx	116.4	59.5	106.0	797.1	−1.0	3.7	HC(W)
1448877_at	Dlx2	17.7	9.0	36.5	119.8	−1.0	3.7	INL+GCL(d)
1449434_at	Car3	11.9	53.5	11.3	700.0	2.2	3.7	AM(G)
1449254_at	Spp1	5.3	35.8	2.0	412.2	2.7	3.5	
1428184_at	3110035E14Rik	66.3	21.5	45.4	237.6	−1.6	3.5	
1460009_at	Ier5	22.6	43.7	32.4	442.5	1.0	3.3	AM+PR+GCL(G)
1436364_x_at	Nfix	87.8	376.3	82.3	3784.4	2.1	3.3	BP+GCL(G)
1442214_at	AK141047	59.0	63.3	44.6	635.9	0.1	3.3	
1437156_at	Necab1	4.0	26.5	3.6	264.5	2.7	3.3	
1456261_at	Sh3kbp1	5.4	11.4	4.8	109.2	1.1	3.3	
1448891_at	Fcrls	3.9	12.9	6.0	122.4	1.7	3.2	
1434777_at	Mycl1	108.0	28.2	100.9	264.9	−1.9	3.2	
1435165_at	Cntn2	9.0	20.5	25.9	186.8	1.2	3.2	
1416034_at	Cd24a	580.5	176.8	592.6	1602.4	−1.7	3.2	MG(B)
1449130_at	Cd1d1	150.3	40.8	116.0	367.1	−1.9	3.2	
1447020_at	AK042819	2.8	11.6	2.5	103.1	2.1	3.1	
1438072_at	AK042460	63.3	77.1	45.6	662.9	0.3	3.1	
1453326_at	3300001A09Rik	26.3	13.0	6.7	110.5	−1.0	3.1	
1457261_at	A930025H08Rik	4.5	12.0	4.9	100.6	1.4	3.1	
1439808_at	A130090K04Rik	19.1	24.6	28.9	204.9	0.4	3.1	
1448288_at	Nfib	33.4	41.4	24.3	333.9	0.3	3.0	
1448194_a_at	H19	191.6	47.4	188.1	379.1	−2.0	3.0	
1457227_at	AI843755	8.9	26.3	7.2	207.1	1.6	3.0	

PR, photoreceptor cell; AM, amacrine cell; BP, bipolar cell; MG, Müller glia; GCL, cells in ganglion cell layer; HC, horizontal cell; INL, cells in inner nuclear layer.

d, de Melo et al., 2003; W, Wakabayashi et al., 2008; B, Blackshaw et al., 2004; G, GENSAT database.

In contrast, the expression profile of strongly up-regulated genes was grouped into two clusters (Fig. 2B, Table 2). Most of members in the first group (15 genes) were down-regulated in the P12 control retina compared to that of P1. This cluster includes transcription factors *Dlx1, Dlx2, Sox11* and *Mycl1*. These genes are possibly involved in several retinal development events such as proliferation of progenitor cells, maturation of early photoreceptor precursors, and cell fate determination of progenitor cells. Consistent with this result, more probes categorized in “cell cycle” were observed in the up-regulated groups (8% at P1 and 4% at P12) compared to those in the down-regulated groups (2% at both P1 and P12) (Fig. 1). Most of members in the second cluster (22 genes) were up-regulated in the P12 control retina compared to that of P1. This group includes *Car3, Spp1, Cst7* and *Col12a1*. We suppose that these genes encode components associated with the increase of amacrine-like cells in the *Otx2* CKO retina.

To determine whether up- or down-regulated genes in the *Otx2* CKO retina are indeed enriched for sets of genes expressed selectively in individual retinal cell subtypes, we performed a statistical analysis. We compared our microarray data sets with previously reported cellular expression patterns of each retinal gene obtained from published *in situ* hybridization data [37]. Forty-five of 84 probes corresponding to photoreceptor-specific genes previously identified by *in situ* hybridization analysis were found in the group down-regulated in the *Otx2* CKO retina (1049 probes; signal log ratio ≤-1.0, signal intensity ≥50). Twenty-one of 70 probes corresponding to amacrine-specific genes previously identified by *in situ* hybridization analysis were found in the group up-regulated in the *Otx2* CKO retina (4149 probes; signal log ratio ≥+1.0, signal intensity ≥50). This data shows that photoreceptor-specific genes are strongly enriched in the group down-regulated in P12 *Otx2* CKO (P<0.01), whereas, the amacrine-specific genes are enriched in the group up-regulated in the P12 *Otx2* CKO retina (P<0.01).

Furthermore, we compared our microarray data with previously reported cellular expression patterns of each retinal gene obtained from published results of *in situ* hybridization [37], [38], immunohistochemistry [39], [40], and cell type–specific GFP expression in the retina using BAC transgenic mice from the GENSAT project [41] (Table 1 and 2). In addition, we searched cone- and rod-specific genes based on the microarray data from *Nrl* KO mice [42] (signal log ratio ≤−2.0 for “down” or ≥+2.0 for “up”). Most of the down-regulated genes in P12 *Otx2* CKO were known cone, rod or pan-photoreceptor genes (Table 1), whereas, several genes up-regulated in the *Otx2* CKO retina were known amacrine- or INL-expressed genes. Expression patterns of many genes in the latter group were previously unidentified in the retina (Table 2).

**Figure 4:**
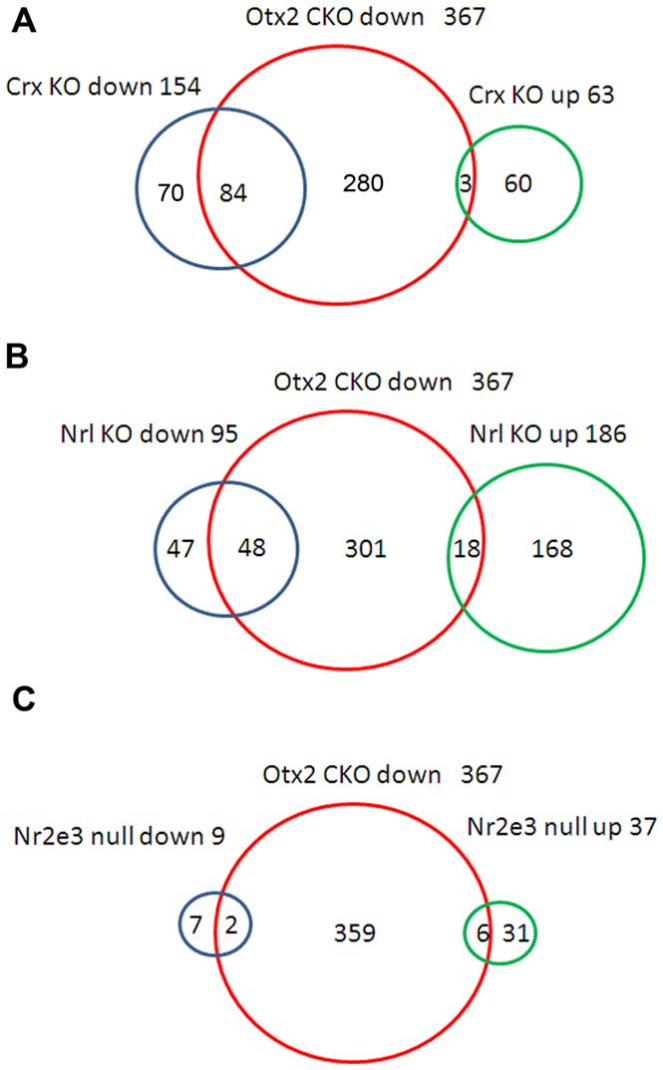
Comparison gene expression profiles with the *Otx2*-CKO retina. (A-C) Microarray analysis datasets from *Crx* (A), *Nrl* (B), *Nr2e3* (C) KO retinas were compared with that from the *Otx2* CKO retina at P12. We identified 367 down-regulated probes in the *Otx2* CKO retina (signal log ratio ≤−2.0, signal intensity ≥74). We used datasets from previous microarray analysis of the *Crx*, *Nrl* and *Nr2e3*-null retinas at P21 with the following results: 154 probes down-regulated in the *Crx* KO retina (signal log ratio ≤−2.0, signal intensity ≥352), 63 probes up-regulated in the *Crx* KO retina (signal log ratio ≥+2.0, signal intensity ≥345), 95 probes down-regulated in the *Nrl* KO retina (signal log ratio ≤−2.0, signal intensity ≥354), 186 probes up-regulated in the *Nrl* KO retina (signal log ratio ≥+2.0, signal intensity ≥347), 9 probes down-regulated in the *Nr2e3*-null (rd7) retina (signal log ratio ≤−2.0, signal intensity ≥354), 37 probes up-regulated in the *Nr2e3*-null retina (signal log ratio ≥+2.0, signal intensity ≥358). The numbers of probes in each category were indicated.

Expression profiles of *Crx*, *Nrl,* and *Nr2e3*-null retinas were reported previously [42], [43], [44], [45]. We compared datasets of the *Otx2* CKO retina with those of the *Crx, Nrl,* and *Nr2e3*-null retinas (Fig. 4, Text S1). We found that the expression of 84 probes was strongly decreased in both the *Otx2* CKO and *Crx* KO retinas (signal log ratio ≤−2.0, Fig. 4A). This group includes both rod and cone photoreceptor genes such as *Rhodopsin* (*Rho*) and *S-opsin* (*Opn1sw*). The expression of 48 probes was markedly decreased in both the *Otx2* CKO and *Nrl* KO retinas (signal log ratio ≤−2.0, Fig. 4B). These genes include rod-specific genes such as *Pde6b* and *Rho*. We identified 18 probes that were down-regulated in the *Otx2* CKO retina but up-regulated in the *Nrl* KO retina, including cone photoreceptor genes such as *Opn1sw*, *cone arrestin (Arr3*) and *Pde6c* (Fig. 4B). Five of six genes down-regulated in the *Otx2* CKO retina but up-regulated in the *Nr2e3*-null retina overlapped with the probes down-regulated in the *Otx2* CKO and up-regulated in the *Nrl* KO retina (Fig. 4C).

### *Otx2* regulates expression of transcription factors involved in retinal development

**Figure 5:**
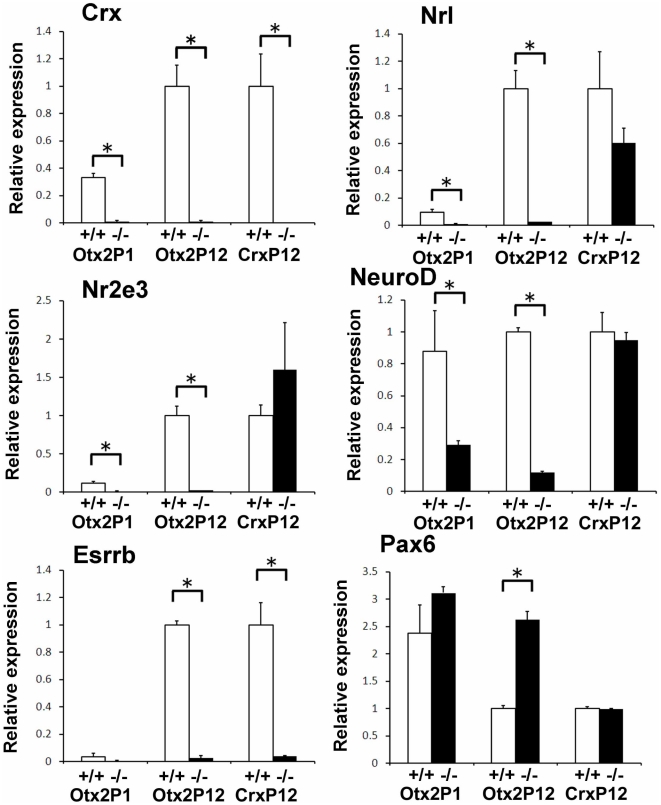
*Otx2* regulates expression of transcription factors involved in retinal development. Expression levels of selected transcription factor genes in the control (at P1 and P12), *Otx2* CKO (at P1 and P12) and *Crx* KO (at P12) retinas were analyzed by Q-PCR. Expression levels of selected genes were normalized to the expression levels of a housekeeping gene, *Gapdh*. The mean of the value of each control at P12 was set as 1.0. Error bars show the SD (n  =  3). *, P<0.03.

To investigate the transcriptional network of *Otx2*-regulated genes, we first focused on the expression of transcription factors involved in retinal development. We previously showed that *Crx* expression was absent in the *Otx2* CKO retina at E18.5, however, the expression of other transcription factors involved in photoreceptor development was not determined [4]. We selected the microarray data sets of 28 transcription factors known to be involved in retinal development (Table 3), and found that the expression of several transcription factor genes (*Crx, Nrl, Nr2e3, Esrrb, Isl1, Blimp1, Pias3* and *NeuroD*) was strongly reduced at P12 (signal log ratio ≤−2.1), whereas the expression level of *Pax6* was increased consistent with the previous result by immunohistochemical analysis [4], [46](Table 3). In addition, we found that the expression of *Crx, Nrl* and *Nr2e3* was strongly reduced at P1 as well (signal log ratio ≤−3.7, Table 3). To validate these microarray results, we carried out quantitative real-time RT-PCR analysis (Q-PCR) for these genes. The Q-PCR results clearly reflect the changes observed in the microarray analysis for all of the genes we tested (Fig. 5).

**Table 3:** Expression of transcription factors involved in retinal development in the *Otx2* CKO retina.

Probe ID	Gene symbol	P1 WT	P1 CKO	SLR	P12 WT	P12 CKO	SLR
1418705_at	Crx	253.7	15.6	−4.0	2932.7	65.3	−5.5
1423631_at	Nr2e3	95.0	7.2	−3.7	2540.0	57.4	−5.5
1450946_at	Nrl	293.9	18.6	−4.0	6073.3	191.9	−5.0
1436926_at	Esrrb	5.1	3.3	−0.6	632.0	24.3	−4.7
1450723_at	Isl1	196.5	219.1	0.2	1542.2	243.0	−2.7
1420425_at	Prdm1/Blimp1	202.4	23.0	−3.1	188.5	34.4	−2.5
1426413_at	NeuroD	296.1	96.7	−1.6	1817.2	333.7	−2.4
1451115_at	Pias3	59.1	24.7	−1.3	860.0	199.7	−2.1
1419628_at	Chx10	423.7	411.8	0.0	2082.0	927.8	−1.2
1424034_at	Rora	49.2	33.8	−0.5	411.0	291.4	−0.5
1418558_at	Rax	168.4	117.3	−0.5	309.0	222.1	−0.5
1418782_at	Rxrg	24.7	17.3	−0.5	41.1	32.7	−0.3
1422202_at	Thrb	4.8	2.6	−0.9	40.9	34.0	−0.3
1450796_at	Math5	32.8	21.4	−0.6	3.2	3.1	0.0
1455799_at	Rorb	465.0	387.2	−0.3	747.8	765.4	0.0
1418102_at	Hes1	111.4	94.5	−0.2	90.1	140.0	0.6
1437588_at	Brn3b/Pou4F2	96.1	123.3	0.4	155.9	284.4	0.9
1427523_at	Six3	190.5	181.7	−0.1	299.7	568.2	0.9
1421336_at	Prox1	18.6	22.8	0.3	199.1	414.2	1.1
1419408_at	Six6	237.1	238.1	0.0	335.8	774.0	1.2
1418633_at	Notch	63.7	61.7	0.0	57.4	141.2	1.3
1418054_at	Math3	165.5	47.0	−1.8	504.4	1277.6	1.3
1419271_at	Pax6	723.9	723.6	0.0	1042.4	3083.1	1.6
1423146_at	Hes5	145.0	155.6	0.1	46.7	157.4	1.8
1422839_at	Neurog2	216.2	277.0	0.4	48.4	296.6	2.6
1437086_at	Mash1	392.6	266.4	−0.6	15.9	119.4	2.9
1448877_at	Dlx2	17.7	36.5	1.0	9.0	119.8	3.7
1449470_at	Dlx1	26.4	66.0	1.3	0.7	273.4	8.6

To compare expression of the transcription factor genes examined with those in the *Crx* KO retina, we analyzed their expression level in the P12 *Crx* KO retina by Q-PCR. In contrast to the drastic decrease of expression in the *Otx2* CKO retina, the expression of most of the transcription factors examined showed only a minor change in the *Crx* KO retina at P12 compared to that of the control retina from wild-type *129/SvEv*. The only notable exception was *Esrrb* which was strongly down-regulated in the *Crx* KO (Fig. 5) [43]. Similar to these transcription factors, we found that the expression changes in genes encoding ciliary and ribbon synaptic components in the *Crx* KO retina were milder than those in the *Otx2* CKO retina (Fig. 3).

### Identification of retinal disease candidate genes

Human homolog mutations of many genes with photoreceptor-associated expression have been shown to be associated with retinal diseases including RP [27]. Since many of the *Otx2* CKO down-regulated genes at P12 were photoreceptor-associated genes (Table 1), we supposed that mutations of human homologs of these down-regulated genes may be responsible for retinal degeneration diseases. To identify retinal disease candidate genes, we determined the chromosomal loci of human homologs of these down-regulated genes and compared these loci with the mapped loci of various hereditary retinal diseases (RetNet, the Retinal Information Network, http://www.sph.uth.tmc.edu/RetNet/). We found that three human retinal disease loci were mapped in close proximity to the markedly down-regulated genes in the *Otx2* CKO retina. Human *CCDC126* is located on 7p15.3 where dominant cystoid macular dystrophy has been mapped [47]. Human *PITPNM1* is located on 11q13 where autosomal dominant neovascular inflammatory vitreoretinopathy was reported [48].

**Figure 6:**
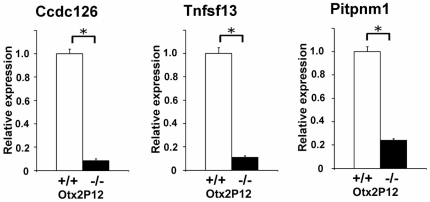
Expression of identified candidate genes for human retinal diseases in the *Otx2* CKO retina. Expression of identified candidate genes for human retinal diseases was analyzed by Q-PCR. Expression levels of selected genes were normalized to the expression levels of a housekeeping gene, *Gapdh*. The mean of the value of control at P12 was set as 1.0. Error bars show the SD (n  =  3). *, P<0.03.

Central areolar choroidal dystrophy was confined to a critical region, an interval of approximately 2.4 Mb (5 cM) flanked by polymorphic markers *D17S1810* and *CHLC GATA7B03* on chromosome 17p13 [49]. Human *TNFSF13* is located between these intervals. The photoreceptor-enriched expression pattern and genomic localization of *Tnfsf13* have been previously reported [37], [38]. *Ccdc126*, *Tnfsf13* and *Pitpnm1* were strongly down-regulated in the *Otx2*-CKO retina, showing signal log ratios of −3.5, −3.0 and −2.4, respectively. We confirmed the decreased expression of these genes in the *Otx2*-null retina by Q-PCR analysis (Fig. 6).

## Discussion

### Otx2 regulates the transcriptional network in photoreceptor development

The main goal of this study is to obtain information and gain insights into the transcriptional network in photoreceptor development regulated by Otx2. Thus, we examined the retinal gene expression profiles of the *Otx2* CKO retina at P1 and P12, and identified significant changes in the expression of genes encoding transcription factors, components of the cilium and ribbon synapse. Our microarray analysis and Q-PCR validation of the *Otx2* CKO retina revealed strong down-regulation of multiple transcription factors involved in photoreceptor development, including *Crx, Nrl, Esrrb, NeuroD, Isl1, Blimp1, Pias3* and *Nr2e3*. These data support the hypothesis that Otx2 organizes a transcription factor network in photoreceptor development. A previous study showed that a considerable amount of *Nrl* expression remains in the *Crx* KO retina, and, similarly, *Crx* expression remains in the *Nrl* KO retinas [43], [44], suggesting that Otx2 regulates the expression of *Nrl* and *Crx* by parallel pathways. Otx2 directly regulates *Crx* expression through binding to cis-regulatory elements on the Crx promoter region [4]. Does Otx2 directly regulate other transcription factors involved in photoreceptor development? The 2.4 kb fragment in the 5′ region of the *Nrl* locus is essential for its expression in photoreceptors and contains Crx/Otx2 binding sites [43]. Since Otx2 and Crx can bind to the same DNA consensus sequence [5], [50], it is possible that Otx2 binds to these cis-regulatory elements and directly regulates *Nrl* expression. In contrast, we found that *Esrrb* expression is almost abolished in both the *Otx2* CKO and the *Crx* KO retinas, showing that *Esrrb* is likely to be regulated directly by Crx and indirectly by Otx2. Similarly, *Nr2e3* expression in the *Nrl* KO was almost abolished [12], suggesting that *Nr2e3* expression is directly regulated by Nrl and indirectly regulated by Otx2. We found that expression of *Isl1, NeuroD, Blimp1,* and *Pias3* was markedly decreased in the P12 *Otx2* CKO retina, however, considerable amounts of expression remained in the *Nrl* KO and *Crx* KO retinas [43]. This result suggests that *Isl1, NeuroD, Blimp1,* and *Pias3* are the direct targets of Otx2. We searched for the Otx2/Crx-binding consensus sequence “TAATC” within 5 kb of the 5′ upstream region of each gene, and found that each of the 5′ regions of *Isl1, NeuroD* and *Pias3* genes contains clusters of the Otx2/Crx-binding sequence (−2.3 to −2.7 kb, 5 sites for *Isl1*; −4.2 to −4.9 kb, 4 sites for *NeuroD*; and −1.3 to −2.0 kb, 4 sites for *Pias3*). Otx2 may directly regulate expression of these transcription factors through these Otx2/Crx-binding sequence clusters. In contrast to the down-regulation of transcription factors involved in photoreceptor development, we observed a 3.0-fold increase of Pax6 expression in the P12 *Otx2* CKO retina. This is probably due to the increase of the amacrine cell population, since amacrine cell markers including *Glyt1* and *Gad65* also increased in the *Otx2* CKO at a similar level (3.0-fold increase for *Glyt1*, 3.5-fold increase for *Gad65*). The result from microarray analysis is consistent with that from immunohistochemical analysis of the *Otx2* CKO retina [4].

We found that photoreceptor-specific genes are strongly enriched in the group of down-regulated probes in the P12 *Otx2* CKO retina, whereas amacrine-specific genes are enriched in the group of up-regulated probes in the P12 *Otx2* CKO retina. Although many of down-regulated genes in the P12 *Otx2* CKO retina were known photoreceptor genes, the expression patterns of several genes, including BC027072/C2orf71, LOC100048701, A930003A15Rik, and 2610034M16Rik, have not been analyzed in the retina. These genes are strong candidates for photoreceptor-specific genes. Expression of 2610034M16Rik was decreased in the *Nrl* KO retina, suggesting that this gene is expressed in rod photoreceptors. In contrast to the high enrichment of photoreceptor genes in the *Otx2* down-regulated probes, amacrine genes were enriched in the group up-regulated in the *Otx2* CKO retina. This group seems to contain not only amacrine associated genes but also various genes from cell types including Muller glia and cells in ganglion cell layer. This result may reflect the increase of aberrant “amacrine-like cells” in the *Otx2* CKO retina.

We found that genes encoding synaptic components (e.g. *CaBP4, Cacna1f* and *Pikachurin*) and ciliary components (e.g. *Rpgrip1* and *Rp1*) were also significantly downregulated in the *Otx2* CKO retina. We previously demonstrated that rhodopsin- and Crx-positive cells were absent in the *Otx2* CKO retina [4], however, there is a possibility that the increased amacrine-like cells converted from photoreceptors in the *Otx2* CKO retina still express photoreceptor-related molecules. Our findings in the present study excluded this possibility and support the idea that Otx2 executes a genetic program on photoreceptor cell fate determination and differentiation. Thus, the transcriptional profile data in this study will be a useful resource to identify genes involved in development and maintenance of both rods and cones.

Previously, we showed that *Otx2* is essential for bipolar cell development [51]. Consistent with this, we observed significant decreases in bipolar genes, including *Bhlhb4, Chx10, Cabp5* and *Pcp2,* in the *Otx2* CKO retina.

The morphological features of rod and cone photoreceptor ribbon synaptic terminals are different; rod photoreceptors form small synaptic terminals with a single ribbon, whereas, cone photoreceptors form larger terminals containing several ribbons with a shorter active zone [24]. These structural differences might be derived from differences of synaptic components. Transcription factors involved in photoreceptor terminal differentiation such as Nrl and Nr2e3 may regulate the expression of rod- or cone-specific synaptic components and contribute to the formation of different structures between cone and rod photoreceptors. Compared with the previous microarray studies, we identified 48 down-regulated probes in both the *Otx2* CKO and *Nrl* KO retinas, while at the same time, 18 probes were down-regulated in the *Otx2* CKO but up-regulated in the *Nrl* KO retina. These data can be a useful resource for finding different mechanisms between cone and rod photoreceptor formation, including ribbon synapse structures.

### Identification of retinal disease candidate genes from *Otx2* downstream genes

The expression profiles of several transcription factors which were shown to be critical for photoreceptor terminal differentiation have been analyzed by microarray or SAGE analysis. The expression profile of the *Crx* KO retina was analyzed using both cDNA microarray and SAGE [38], [43], [52]. Analyses of the expression profiles of *Nrl* KO, *Nr2e3* KO or *Nrl* & *Nr2e3* double KO retinas were performed using microarrays [42], [43], [44], [45]. Lack of *Crx* does not affect photoreceptor cell fate but does result in abnormal photoreceptor morphogenesis [7]. Lack of either *Nrl* or *Nr2e3* causes photoreceptor subtype conversion from rod photoreceptor to S-cone photoreceptors [12], [14]. Since deletion of *Otx2* leads to a total loss of photoreceptors in the retina, by comparing expression profiles between the *Otx2* CKO retina and control retina, we were able to identify photoreceptor-associated genes more clearly than by using other mutant retinas. Comparing the expression profiles between the *Otx2* CKO and other mutant mice may provide novel insight into genetic transcriptional networks in photoreceptor development. Furthermore, we identified several retinal disease candidate genes among the genes down-regulated in the *Otx2* CKO retina based on information from RetNet's mapped retinal disease loci. Linking information from other databases to the set of *Otx2* down-regulated genes presented in our study may give unexpected insights into photoreceptor biology. For example, proteome analysis of purified photoreceptor sensory cilium revealed that this complex contains 1,968 proteins [53]. By comparing our data and the data from other studies, a novel insight on the mechanisms of retinal disease might be obtained in the future.

## Materials and Methods

### Animals

The *Otx2* CKO and *Crx* KO mice were generated in our previous studies [4], [7]. All procedures conformed to the ARVO statement for the Use of Animals in Ophthalmic and Vision Research, and these procedures were approved by the Institutional Safety Committee on Recombinant DNA Experiments and the Animal Research Committee of Osaka Bioscience Institute (approval ID 10-401). Mice were housed in a temperature-controlled room with a 12 h light/dark cycle. Fresh water and rodent diet were available at all times.

### Microarray profiling

The P1 or P12 retinas of mouse were dissected. Total RNA (5 µg) of the retina was isolated using TRIzol reagent (Invitrogen) and converted to cDNA using One-Cycle cDNA synthesis kit (Affymetrix) according to the manufacture's instruction. Biotin-labeled cRNA was prepared using IVT labeling kit and hybridized to GeneChip mouse genome 430 2.0 array (Affymetrix). Signal intensity was determined using GeneChip Operating Software 1.4. Microarray expression data are MIAME compliant and have been deposited in a MIAME compliant database (GEO accession number GSE21900).

### Q-PCR

The P1 or P12 retinas of mouse were dissected. Total RNA (1 µg) of the retina was isolated using TRIzol reagent (Invitrogen) and converted to cDNA using Superscript II RTase (Invitrogen). Real time qPCR was performed using Cyber Green ER qPCR Super MIX (Invitrogen) and Thermal Cycler Dice Real Time System single MRQ TP870 (Takara) according to the manufacture's instruction. Quantification was performed by Thermal Cycler Dice Real Time System software Ver. 2.0 (Takara). To amplify the gene fragments, we used primers as listed in Table S1.

## Supporting Information

Table S1 Primers for Q-PCR analysis.
(XLS)

Text S1 Probe IDs and gene symbols for each group in Figure 4.
(DOC)
