# Supplementary material for: Analysis of Transcriptional Regulatory Pathways of Photoreceptor Genes by Expression Profiling of the Otx2-Deficient Retina
Source: PLoS One. 2011 May 13;6(5):e19685. doi: 10.1371/journal.pone.0019685 (PMC3094341; doi:10.1371/journal.pone.0019685)
Supplement: Text S1 — Probe IDs and gene symbols for each group in Figure 4. (DOC) [file pone.0019685.s002.doc]

**Text S1**

**Probe IDs and gene symbols for each group in Figure 3:**

***Otx2*-CKO-down & *Crx*-KO-down (84 probes)**

1454209_at,Fam57b;1430112_at,Wdr66;1437371_at,Fam160a1;1450789_at,Rhpn1;1443232_at,Vax2os2;1431357_a_at,Rpgrip1;1418552_at,Opn1sw;1440605_at,Fscn2;1444404_at,Rnf207;1420372_at,Sntb2;1442242_at,C79127;1425919_at,Ndufa12;1422986_at,Esrrb;1460479_at,A330094K24Rik;1459664_at,Wdr31;1425696_at,Nxnl1;1444230_at,2900046D03Rik;1439150_x_at,Grtp1;1446754_a_at,Vax2os1;1426035_at,RP23-433P19.11;1423636_at,Wdr31;1423611_at,Alpl;1435189_at,Frmpd1;1425891_a_at,Grtp1;1450829_at,Tnfaip3;1453422_a_at,1110020G09Rik;1456905_at,Ccdc24;1450816_at,Polg2;1428975_at,Susd3;1451785_at,Rpgrip1;1416203_at,Aqp1;1454231_a_at,Rpgrip1;1457083_at;1444095_a_at,LOC100048701;1458418_at,Gm2380;1449421_a_at,Kcne2;1436277_at,Rnf207;1425878_at,Cabp4;1456936_at,Cabp4;1419647_a_at,Ier3;1448169_at,Krt18;1428680_at,Cds1;1436402_at,Dohh;1425408_a_at,2610034M16Rik;1421085_at,Rs1;1458506_at,Gm626;1451636_at,Ankrd33;1451210_at,Ppap2c;1449588_at,Abca4;1424895_at,Gpsm2;1421144_at,Rpgrip1;1436926_at,Esrrb;1450765_a_at,Pde6h;1451549_at,BC016201;1417430_at,Cdr2;1421084_at,Rs1;1419723_at,Opn1mw;1455098_a_at,Vtn;1450766_at,Pde6h;1431010_a_at,Rdh12;1429133_at,Nxnl2;1433699_at,Tnfaip3;1423179_at,Kcnb1;1441330_at,Crb1;1423180_at,Kcnb1;1431174_at;1440256_at,Rgs9bp;1436657_at,Gm11744;1452243_at,Kcnj14;1425138_at,Guca1b;1451603_at,Rtbdn;1424256_at,Rdh12;1449132_at,Opn1sw;1420484_a_at,Vtn;1430128_a_at,Reep6;1451647_at,Slc24a1;1450215_at,Rcvrn;1450453_a_at,Pde6g;1425100_a_at,Pde6g;1417432_a_at,Gnb1;1451617_at,Rho;1460212_at,Gnat1;1425171_at,Rho;1451618_at,Rho

***Otx2*-CKO-down & *Crx*-KO-up (3 probes)**

1454770_at,Cckbr;1417889_at,Apobec2;1450779_at,Fabp7

***Otx2*-CKO-down & *Nrl*-KO-down (48 probes)**

1437371_at,Fam160a1;1422986_at,Esrrb;1458177_at,Ebpl;1443232_at,Vax2os2;1425441_at;1450829_at,Tnfaip3;1418478_at,Lmo1;1446754_a_at,Vax2os1;1440605_at,Fscn2;1416203_at,Aqp1;1417288_at,Plekha2;1436277_at,Rnf207;1431174_at;1450816_at,Polg2;1451210_at,Ppap2c;1428975_at,Susd3;1428547_at,Nt5e;1449945_at,Ppargc1b;1455140_at,Pitpnm3;1458418_at,Gm2380;1423631_at,Nr2e3;1425408_a_at,2610034M16Rik;1450415_at,Pde6a;1436926_at,Esrrb;1424895_at,Gpsm2;1417430_at,Cdr2;1451647_at,Slc24a1;1433699_at,Tnfaip3;1423180_at,Kcnb1;1451549_at,BC016201;1452243_at,Kcnj14;1425288_at,Samd11;1417298_at,Ebpl;1440838_at;1423179_at,Kcnb1;1451763_at,Cnga1;1452806_at,Fam57b;1417432_a_at,Gnb1;1430128_a_at,Reep6;1425138_at,Guca1b;1452807_s_at,Fam57b;1419740_at,Pde6b;1451617_at,Rho;1450946_at,Nrl;1451618_at,Rho;1425171_at,Rho;1460212_at,Gnat1;1425172_at,Rho

***Otx2*-CKO-down & *Nrl*-KO-up (18 probes)**

1419099_x_at,Stom;1453127_at,Ppm1j;1422907_at,Gnat2;1450329_a_at,Arr3;1423636_at,Wdr31;1449421_a_at,Kcne2;1417396_at,Podxl;1460605_at,Crxos1;1442160_at,Fam19a3;1425232_x_at,Arr3;1450830_a_at,Pde6c;1438399_at,Pex5l;1449501_a_at,Gzmm;1421780_a_at,Cabp5;1454770_at,Cckbr;1429036_at,Otop3;1418552_at,Opn1sw;1450779_at,Fabp7

***Otx2*-CKO-down & *Nr2e3*-null-down (2 probes)**

1416203_at,Aqp1;1417889_at,Apobec2

***Otx2*-CKO-down & *Nr2e3*-null-up (6 probes)**

1422907_at,Gnat2;1454770_at,Cckbr;1448602_at,Pygm;1450830_a_at,Pde6c;1429036_at,Otop3;1450779_at,Fabp7
